# How Is the Relationship of Spiritual Health and Body Image with the Desire for Aesthetic Surgery among Students?

**DOI:** 10.29252/wjps.9.2.225

**Published:** 2020-05

**Authors:** Mokhtar Soheylizad, Younes Mohammadi, Elahe Ezati, Babak Moeini

**Affiliations:** 1 Student Research Committee, Hamadan University of Medical Sciences, Hamadan, Iran;; 2 Modeling of Non-communicable Diseases Research Center, Hamadan University of Medical Sciences, Hamadan, Iran;; 3 Social Determinants of Health Research Center, Hamadan University of Medical Sciences, Hamadan, Iran

**Keywords:** KEYWORDS

## Abstract

**BACKGROUND:**

The desire for aesthetic surgery in Iran has increased. The relationship between spirituality and body image has not been studied simultaneously with the desire for aesthetic surgery. The present study aimed to examine this relationship among students of Hamadan University of Medical Sciences, Hamadan, Iran.

**METHODS:**

In this analytical cross-sectional study in 2019, 397 students were enrolled by stratified random sampling. The data were collected using the Paloutzian and Ellison’s spiritual health questionnaire and Appearance Schemas Inventory. Data were analyzed using Pearson correlation, independent t-test and logistic regression.

**RESULTS:**

The mean age of the subjects was 22.26±4.24 years, while 26.7% of the subjects had desire for aesthetic surgery. There was a significant negative correlation between body image and spiritual health (*p*<0.001). The mean score of spiritual health and its dimensions in female students were higher than males. Based on logistic regression model, age (*p*=0.018, 95% CI: 0.341-0.904) and body image (*p*<0.001, 95% CI: 1.05-1.112) had significant correlation with the desire for aesthetic surgery.

**CONCLUSION:**

According to the results; it is necessary to make plans for the promotion of spirituality and the strengthening of a positive body image among students residing in student homes, male students and those who desire to engage aesthetic surgery.

## INTRODUCTION

The desire to beauty from the distant past has always been with human^1^ and is considered a high value for beauty in human societies; this has led many people to perform various types of aesthetic surgery for increasing the attractiveness.^2^ Some of the aesthetic surgeries include liposuction, reshaping and resizing of the nose and eyelids; breast augmentation, laser hair removal, face lift, as well as minimally-invasive procedures like Botox and chemical peels.^3^ In 2016, more than 23 million aesthetic surgeries have been performed worldwide, that seems to be up from 9% in 2015.^4^

According to the American Society of Plastic Surgeons (ASPS), the world’s largest organization of board-certified plastic surgeons, aesthetic surgery is very common in the United States; in 2018, nearly 18 million surgeries have been performed in US, that shows a rise of about 2 percent from 2017.^5^ Although there is no official statistics on the prevalence of aesthetic surgeries in Iran,^6^ the results of some studies indicated that aesthetic surgeries in Iran have increased over the past decades.^7^

The extreme tendency of the community to various types of aesthetic surgery will subsequently lead to inflict of vast materials and spiritual costs for individuals, families and, ultimately, the community.^8^ The results of various studies have shown that individuals undergoing aesthetic surgery have experienced a variety of psychological disorders.^8^^,^^9^ Having a negative body image is one of the factors that leads people to aesthetic surgery.^10^ The body image is an abstract concept that refers to the perception of individuals from the appearance of the body, and this concept includes emotional, cognitive, behavioral, and perceptual aspects.^11^

The body image can have a dramatic effect on how people perceive the world around them and affect other aspects of life, such as self-esteem, mood, nutritional behavior, and social interactions.^12^ Repeating negative perceptions of the body may lead to a body dysmorphic disorder (BDD).^13^ As mentioned, one of the consequences of performing aesthetic surgery is heavy spiritual costs on individuals, families and society.^8^ By compromising spiritual health, a person may experience psychiatric disorders such as loneliness, depression and loss of meaning in life;^14^ while applicants for aesthetic surgery are confronted with such mental disorders.^15^

Spiritual health, as the newest dimension of health, is linked to other dimensions of health and causes other dimensions to be integrated.^16^ Spiritual health has a positive relationship with the physical and mental dimensions of quality of life. Therefore, spiritual health is associated with quality of life and, on the other hand, quality of life results from satisfaction or dissatisfaction from various aspects such as status of appearance, social relations and so on.^17^ So considering the importance of spirituality and its important role in the health and life of individuals, families and consequently the community; in addition to the body image, another issue that has been investigated in this study is spiritual health.

Considering that few studies have been conducted for explaining factors associated with aesthetic surgery in young people, especially male and female students; a perfect research is required in order to investigate, study and explain the role of multiple factors such as body image and spiritual health in desire for aesthetic surgery. Therefore, the present study was conducted to explain the relationship between spiritual health and body image with the desire for aesthetic surgery among students of Hamadan University of Medical Sciences.

## MATERIALS AND METHODS

This analytical cross-sectional study was conducted among 397 students of Hamadan University of Medical Sciences, west of Iran, at the beginning of 2019. To estimate the sample size, according to study of Tahmasebi *et al.*,^18^ the desire for aesthetic surgery was 50% (*p*=0.5), 95% confidence interval and accuracy of 5% (d=0.05). By putting these values in the following formula, the sample size was 384. Considering the probability of incomplete questionnaires, the final sample size was 400.

$n=\frac{Z_{1-\alpha /2}^{2}\times p(1-p)}{d^{2}}=\frac{3.8416\times \left(0.5\times 0.5\right)}{0.0025}=384$

Sampling was done by stratified, proportional allocation and random sampling. Each college was considered as a class, and then, according to the number of male and female students of each college and according to the course and the education grade, random sampling was done. The inclusion criteria were being a student at Hamadan University of Medical Sciences and having the consent to participate in the study. The incomplete completion of the questionnaire was considered as exclusion criterion.

To collect the data, the researchers referred to each college and after coordination with each class’s teachers, after expressing the goals of the study and obtaining informed consent from the students to attend the study, the self-administered questionnaires were completed randomly by the students in the classroom. This study was approved by the Ethics Committee of Hamadan University of Medical Sciences with the ethics code: IR.UMSHA.REC.1396.767. The instruments used to collect data were the following questionnaires: (i) Demographic and background information questionnaire.

Data on gender, age, marital status, education grade, permanent and current residence, individual or family monthly income in Rial, national currency of Iran (at the time of the study, every 160,000 Rials was equivalent to $ 1), and history of any type of aesthetic surgery and desire for aesthetic surgery was obtained from this questionnaire. (ii) Appearance Schemas Inventory (ASI): This questionnaire consisted of 14 items that gave a negative opinion of a person about his appearance that acted like a schema, and higher scores represented the negative beliefs of a person about his appearance. 

Scoring was done in the 5-point Likert range, which was rated as 1 (completely disagree) to 5 (completely agree). Higher scores represented more negative beliefs that a person had toward his or her appearance. For the reliability of its Persian form, in the study of Sadeghi *et al.*, the Cronbach’s alpha coefficient, test-retest and split-half were reported as 75%, 82% and 78%, respectively. The validity of this questionnaire was calculated using the Multidimensional Body-Self Relations Questionnaire (MBSRQ) and the correlation was -0.88.^19^ In this study, the reliability of this questionnaire was obtained through Cronbach’s alpha coefficient to be 0.84.

(iii) Spiritual health questionnaire: To measure spiritual health, 20-items Paloutzian and Ellison’s spiritual health questionnaire, which measures 10 items of religious health (odd items) and 10 other items of existential health (even items), was used. Scoring was done in the 7-point Likert range, from “completely agree” to “completely disagree”. In the negative items, the scoring was done in reverse order (items 3, 4, 7, 8, 10, 11, 14, 15, 17, 19, 20 “completely disagree” with score 1; and items 1, 2, 5, 6, 9, 12, 13, 16, 18, “completely disagree” with score 7). 

Finally, spiritual health was divided into three levels: Low as 20-44, medium as 45-115, and high as 116-140. In the study of Fatemi *et al.*, the validity of this questionnaire was confirmed through content validity, and its reliability was determined through the reliability coefficient of alpha Cronbach of 0.82.^20^ In this study, Cronbach’s alpha coefficient was 0.93. After collecting data, incomplete questionnaires were excluded from the study. Finally, 397 questionnaires were analyzed by SPSS software (version 24, Chicago, IL, USA), using Pearson correlation, independent t-test and logistic regression. The significance level of all tests was considered to be less than 0.05.

## RESULTS

The results of the demographic characteristics of the participants in the study were shown in Table 1. As shown in this table, 60.7% of the participants were female. The mean age of the subjects was 22.26±4.24 years and in total, 56.7% of them were under the age of 22 and 88.7% were single. Generally, 55.4% of students were at undergraduate level. Totally, 93.7% of the subjects were permanently resident in urban areas; at the time of the study, 70% of them lived in a dormitory or student home, while the rest were resident in Hamadan city. In terms of income, 27.7.1% of students reported their monthly income between 10 to 20 million Rials per month. 

**Table 1:** Demographic and background characteristics of the subjects

**Variable**		**Frequency**	**Percent (%)**
Gender	Male	156	39.3
	Female	241	60.7
Age groups	<22	225	56.7
	≥22	172	43.3
Marital status	Single	352	88.7
	Married	43	10.8
	Divorced	2	0.5
Education grade	Bachelor	220	55.4
	Doctorate	156	39.3
	Graduate	21	4.3
Permanent residence	Urban	372	93.7
	Rural	25	6.3
Current residence	Dormitory	254	64
	Student home	24	6
	Hamadan	119	30
Income	<10 million	38	9.6
	10-20 million	110	27.7
	20-30 million	109	27.5
	30-40 million	75	18.9
	>40 million	65	16.4
History of aesthetic surgery	Yes	23	5.8
	No	374	94.2
Desire for aesthetic surgery	Yes	106	26.7
	No	291	73.3

Twenty three (5.8%) of the subjects had a history of aesthetic surgery, and 106 (26.7%) had desire for aesthetic surgery. Pearson correlation test results were presented in Table 2. As shown in this table, there was a positive and significant correlation between the existential dimension of spiritual health and religious dimension (*p*<0.001). On the other hand, there was a significant negative correlation between body image and spiritual health (*p*<0.001) as well as religious (*p*<0.05) and existential dimensions (*p*<0.001).

**Table 2:** Correlation between variables of age, spiritual health and body image

**Variable**	**1**	**2**	**3**	**4**	**5**
Age	1				
Religious dimension	0.045	1			
Existential dimension	0.007	0.65^**^	1		
Spiritual health	0.029	0.912^**^	0.904^**^	1	
Body image	0.051	-0.126^*^	-0.206^**^	-0.182^**^	1

*Correlation is significant at the 0.05 level, **Correlation is significant at the 0.01 level

The results of the comparison of the mean score of spiritual health, its dimensions and the body image of the subjects according to the desire for aesthetic surgery were shown in Table 3. The mean score of spiritual health and its existential dimension were lower among those who were willing to perform aesthetic surgery. The mean of body image score among those who desired to have aesthetic surgery and those who did not, were 42.09 and 35.98, respectively. According to the independent t-test, this difference was statistically significant (*p*<0.001).

**Table 3:** Comparison of the mean of spiritual health, its dimensions and body image in terms of the desire for aesthetic surgery

**Variables**	**Desire for aesthetic surgery**		***p*** ** value**
	**Yes**	**No**	
	**Mean±SD**	**Mean±SD**	
Spiritual health	103.68±19.79	105.28±20.52	0.448
Religious dimension	55.57±10.49	55.08±11.74	0.709
Existential dimension	48.11±11.57	50.2±10.68	0.093
Body image	42.09±9.29	35.98±8.59	<0.001

The results of the comparison of the mean score of spiritual health, its dimensions and the body image of the subjects in terms of gender were shown in Table 4. The mean score of spiritual health and its dimensions in female students were higher than males. This difference was statistically significant for spiritual health and religious dimension (*p*<0.001). The mean score of the body image was higher in males, but this difference was not statistically significant (*p*=0.159). To determine the variables affecting the desire for aesthetic surgery, the univariate and then multivariate logistic regression model were used. Based on this model, age (*p*=0.018, 95% CI: 0.341-0.904) and body image (*p*<0.001, 95% CI: 1.05-1.112) had significant correlation with the desire for aesthetic surgery (Table 5).

**Table 4:** Comparison of the mean of spiritual health, its dimensions and body image in terms of gender

**Variables**	**Gender**		***p*** ** value**
	**Male**	**Female**	
	**Mean±SD**	**Mean±SD**	
Spiritual health	100.33±20.54	107.78±19.66	<0.001
Religious dimension	51.91±12.8	57.35±9.87	<0.001
Existential dimension	48.42±10.24	50.44±11.33	0.073
Body image	38.42±8.28	37.09±9.7	0.159

**Table 5:** The effect of predictive variables on the desire for aesthetic surgery

**Variable**		**Univariate**			**Multivariate**		
		**OR (Crude)**	**CI (95%)**	***p*** ** value**	**OR (Adjusted)**	**CI (95%)**	***p*** ** value**
Gender	Female	1 (Reference)					
	Male	0.654	(0.409-1.046)	0.077	0.591	(0.384-1.004)	0.052
Age groups	≥22	1 (Reference)					
	<22						
Current residence	Hamadan	1 (Reference)					
	Dormitory						
	Student home						
Income	>40 million	1 (Reference)					
	<10 million						
	10-20 million						
	20-30 million						
	30-40 million						
History of aesthetic surgery	No	1 (Reference)					
	Yes						
Spiritual health^*^		^**^			^**^		
Religious dimension^*^							
Existential dimension^*^		^**^			^**^		
Body image^*^							

*In order to implement the interpretation of the results, the values of B are multiplied by 10 units and then the odds ratio is calculated. **The sign B has been negative.

.

## DISCUSSION

According to the results of this study, 26.7% of participated students desired for aesthetic surgery; while this finding was not consistent with the results of Dehdari *et al.*, which indicated that 6.61% of the subjects intended to perform aesthetic surgery.^6^ There are several points to note about this disparity; one of these issues is a six-year interval between the two studies; over the years, the factors associated with aesthetic surgery have undergone change in order to increase the desire for aesthetic surgery. On the other hand, the increase in the number of aesthetic surgery performed in Iranian society in recent years confirms this issue.^7^

One of the criteria for entering the mentioned study was “no previous history of aesthetic surgery”, while our study did not have this limitation. The results showed that about half of the subjects with a history of aesthetic surgery in the present study again desired to undergo aesthetic surgery. On the other hand, according to the results of the logistic regression model, the likelihood of having aesthetic surgery in people with a history of aesthetic surgery was more than those who did not have such a history. According to the results of this study, the desire for aesthetic surgery in females was twice as high as that of males; this finding has been reported in other studies.^21^^-^^23^

Women are more concerned about beauty for individual reasons or considering the social conditions, in order to obtain a suitable social status and to promote it. It is necessary to have more beauty and therefore, tending to perform aesthetic surgery more than men. Based on the results of the present study, people over 22 years of age were more likely to have aesthetic surgery than those under the age of 22 years. In other words, the desire to perform aesthetic surgery has increased with age. Our finding is consistent with the results of studies on the tendency to perform Botox^24^ and an internet survey to examine the desire for aesthetic surgery.^23^

But our finding not consistent with the results of the study performed among students from different universities in Tehran, Iran.^2^ In order to explain this finding, it can be pointed out that the passage of life is inevitable, and in general; as the age increases, the beauty of the face and body diminishes; consequently, people may believe that the attractiveness as well their popularity and social status will be eliminated. On the other hand, the effect of performing aesthetic surgery on younger age and improving attractiveness, especially in women, has been reported.^2^ Therefore, an increase in the desire for performing aesthetic surgery with an increase in age is expected.

Subjects that lived in student homes at the time of study were more likely to perform aesthetic surgery. To illustrate this finding, there are several points to consider such as students, who were typically at young age, had autonomy morale and want to have freedom to shape their attitudes, intentions and behaviors. This is partly achieved by moving away from the family environment and the pressures, standards, and care that existed there; of course, this freedom of action in student homes was more visible due to less care than student dormitories. In sum, these conditions may lead to desire formation for aesthetic surgery. 

On the other hand, familiarity, companionship and fellowship with people who may have experienced numerous aesthetic surgeries, due to the ease of interaction with others in student homes, occur more often. Iterating the possible positive experiences and feelings of these individuals from the surgeries may affect students’ thoughts, beliefs, and desires toward performing aesthetic surgery. According to the results of this study, those with a higher monthly income were more likely to perform aesthetic surgery. Performing aesthetic surgery involved paying for heavy costs. 

On the other hand, low-income people made other living needs a priority. Therefore, it seems logical that such people to have less tendencies to perform aesthetic surgery. The results of this study showed that there was a negative and significant correlation between body image and spiritual health and its religious and existential dimensions. These results are consistent with the findings of a study in China^25^ and a systematic review study.^26^ Since both religious and existential dimensions are considered for spiritual health, consideration should be given to both dimensions in order to explain this finding.^27^

The religious dimension is defined as a connection and a commitment to a particular religious belief. Religion is one of the main components of the culture of any society that guides it and ensures social cohesion. Religious beliefs, religion, and religious rituals give people peace of mind and guarantee the safety of the individual, strengthen the moral and emotional emptiness of the individual, and establish a solid base for human beings against problems, shortcomings, and deprivations of life.^27^

For some people, one of these shortcomings and deprivations may be appearance dissatisfaction and a negative image. Religious health can prevent psychological stress; and it can also act as a barrier against this inappropriate understanding and make the person accept his appearance as it is; consequently, his body and its shape and appearance will not look like a defect in life. On the other hand, the existential dimension of spiritual health refers to the meaning of life and how to achieve perfection. Therefore, if a person has a desirable level of existential health, he considers his life as purposeful. In order to achieve the ultimate perfection, he will plan accordingly with those goals.^28^

Therefore, a person does not consider his appearance as an effective factor in achieving those goals, and the existence of an abnormality in his appearance does not constitute an obstacle to achieving those goals. The results of the present study showed that those who desired for aesthetic surgery had a more negative perception of their appearance. The logistic regression analysis also confirmed this issue. By increasing the body image score, which indicated a negative image of the body, the chance of having a desire for aesthetic surgery also increased significantly. Regarding this finding, it can be said that those who did not have an appropriate body image and whose perception of their appearance is negative, they considered doing aesthetic surgery as a way of escaping from this conception and negative perception.^28^

Because the negative image is associated with psychological, mood, behavioral, and social disorders, these disturbances lead to annoying experiences for the individuals. Therefore, aesthetic surgery will be a way to overcome these problems. These persons may also consider aesthetic surgery as a solution to surmount their body impairment and to correct their imagination from the body.^12^

Concerning the spiritual health, the results showed that those who desired to perform aesthetic surgery had less mean score of spiritual health. On the other hand, the results of regression analysis showed that with increasing spiritual health as well as its existential dimension, the chance of desire for aesthetic surgery decreased. When individuals had an optimal existential health, they were more compatible with a variety of different situations in life. One of these conditions was the appearance of the body, which such persons may adapt to it, even if there was a likelihood of defect in their appearance, and accepted it along with other living conditions.

As a result, they were less likely to suffer from mental and psychological problems due to defects and possible problems in appearance, and were more satisfied with their appearance; therefore, they were reluctant to perform aesthetic surgery as a way to overcome these concerns. Based on the results of our study, the spiritual health score as well as its religious and existential dimensions was higher in female students than males; and this finding is consistent with the results of numerous studies conducted both in Iran^27^^,^^29^^,^^30^ and other countries.^31^^-^^33^

Differences in the level of spiritual health of women and men may be attributed to their specific characteristics and mental and emotional capacities; women are more adaptable than men, because women take on multiple roles and responsibilities in life, which increased their spiritual compatibility. Another finding of our study was that the mean score of the male body image was higher than that of females. In other words, men had a more negative perception of their bodies than women. This finding is not consistent with the results of studies conducted in US^34^ and Poland.^35^

This contradiction may be due to the difference in the target group of these studies and the differences in cultural and social conditions at the site of these studies. However, considering that the present study was conducted among students, the following can be mentioned. The period of university education is interacting with the opposite sex; due to the limited communication between the boy and the girl in the Iranian society, boys who have a good appearance, if they are successful in communicating with girls, may lead to the formation of this belief in other boys, that this success is due to having the appropriate appearance not having the ability to communicate. 

Therefore, those who fail to establish such communications suppose the cause of this failure is that they do not have the proper appearance, which can lead to the formation of a negative body image among them. On the other hand, the cultural, individual, family and social changes that have occurred during recent years in Iranian society should not be ignored, which, of course, requires further studies in this regard. In this study, in addition to examining the relationship between body image and desire for aesthetic surgery and its related factors, the relationship between spiritual health and its dimensions with body image and also desire for aesthetic surgery was investigated. This can be considered as the strength of this study.

Considering the fact that the present study was conducted among students of Hamadan University of Medical Sciences, the results of this study cannot be generalized to other population groups; and this is considered as a limitation of this study. Therefore, similar studies are recommended in specific population groups and other groups of society. The results of our study emphasized the importance of addressing the spiritual health and body image among students of various medical sciences. Considering the high desire for performing aesthetic surgery among students, it is necessary to make plans for the promotion of spirituality and the strengthening of a positive body image among students residing in student homes, male students and those who desired to perform aesthetic surgery.

## References

[1] Wolpe PR (2002). Treatment, enhancement, and the ethics of neurotherapeutics. Brain Cogn.

[2] Kalantar-Hormozi A, Jamali R, Atari M (2016). Interest in cosmetic surgery among Iranian women: the role of self-esteem, narcissism, and self-perceived attractiveness. Eur J Plast Surg.

[3] Jung J, Hwang CS (2016). Associations between attitudes toward cosmetic surgery, celebrity worship, and body image among South Korean and US female college students. Fashion and Textiles.

[4] International Society of Aesthetic Plastic Surgery ISAPS Global Statistics.

[5] American Society of Plastic Surgeons 2016 Plastic Surgery Statistics.

[6] Dehdari T, Khanipou A, Khazir Z, Dehdari L (2015). Predict the intention to perform cosmetic surgery on female college students based on the theory of reasoned action. Military Caring Sciences Journal.

[7] Mojallal M, Khosrojavid M, Pakzad F, Ghanbari M (2014). Early maladaptive schemas, body image, and self-esteem in iranian patients undergone cosmetic surgery compared with normal individuals. Practice in Clinical Psychology.

[8] Valikhani A, Goodarzi MA (2017). Contingencies of Self-Worth and Psychological Distress in Iranian Patients Seeking Cosmetic Surgery: Integrative Self-Knowledge as Mediator. Aesthetic Plast Surg.

[9] Collins B, Gonzalez D, Gaudilliere DK, Shrestha P, Girod S (2014). Body dysmorphic disorder and psychological distress in orthognathic surgery patients. J Oral Maxillofac Surg.

[10] R YY, Hayatbini N, Asgharnejad Farid AA, Gharaee B, Latifi NA (2016). The Body Image Dissatisfaction and Psychological Symptoms among Invasive and Minimally Invasive Aesthetic Surgery Patients. World J Plast Surg.

[11] Cash TF (2004). Body image: past, present, and future. Body Image.

[12] Nair BP, Baboo G (2017). Effect of Cosmetic Surgery on Body Image and Body Image Specific Quality of Life. Journal of the Indian Academy of Applied Psychology.

[13] Veale D, Gledhill LJ, Christodoulou P, Hodsoll J (2016). Body dysmorphic disorder in different settings: A systematic review and estimated weighted prevalence. Body Image.

[14] Safavi M, Fesharaki M, Oladrostam N, Fatahi Y (2016). An investigation of the relationship between spiritual health and depression, anxiety, and stress in patients with heart failure. Health, Spirituality and Medical Ethics.

[15] Herruer JM, Prins JB, van Heerbeek N, Verhage-Damen GW, Ingels KJ (2015). Negative predictors for satisfaction in patients seeking facial cosmetic surgery: a systematic review. Plast Reconstr Surg.

[16] Ajam Zibad H, Mohammadi Shahboulaghi F, Foroughan M, Rafiey H, Rassouli M (2016). What is the meaning of spiritual health among older adults? A concept analysis. Educational Gerontology.

[17] Dalmida SG (2006). Spirituality, mental health, physical health, and health-related quality of life among women with HIV/AIDS: integrating spirituality into mental health care. Issues Ment Health Nurs.

[18] Tahmasbi S, Tahmasbi Z, Yaghmaie F (2014). Factors related to cosmetic surgery based on theory of reasoned action in shahrekord students. Journal of Holistic Nursing And Midwifery.

[19] Sadeghi K, Gharraee B, Fata L, Mazhari SZ (2010). Effectiveness of cognitive-behavioral therapy in treating patients with obesity. Iranian Journal of Psychiatry and Clinical Psychology.

[20] Fatemi N, Rezaii M, Givari A, Hosini F (2006). Pray for the spiritual health of cancer patients. Payesh Health Monitor, Journal of the Iranian Institute for Health Sciences Research.

[21] Milothridis P, Pavlidis L, Haidich AB, Panagopoulou E (2016). A systematic review of the factors predicting the interest in cosmetic plastic surgery. Indian J Plast Surg.

[22] Babadi H, Fereidooni-Moghadam M, Dashtbozorgi B, Cheraghian B (2018). Investigating Psychosocial Causes of the Tendency for Facial Cosmetic Surgery. Aesthetic Plast Surg.

[23] Frederick DA, Lever J, Peplau LA (2007). Interest in cosmetic surgery and body image: views of men and women across the lifespan. Plast Reconstr Surg.

[24] Kabodi S, Salimi E, Kavoussi H, Ebrahimi A, Ashtaria H, Rajabi Gilan N, Shirzadi M (2017). Role of body image, social support and religious beliefs in predicting botox cosmetic surgery. Journal of Mazandaran University of Medical Sciences.

[25] Zhang KC (2013). What I look like: college women, body image, and spirituality. J Relig Health.

[26] Akrawi D, Bartrop R, Potter U, Touyz S (2015). Religiosity, spirituality in relation to disordered eating and body image concerns: A systematic review. J Eat Disord.

[27] TAVAN H, Taghinejad H, Sayehmiri K, Yary Y, Fathizadeh H, Saraby A, Poorabdollah H (2015). Spiritual health of nursing students. Islam and Health Journal.

[28] R YY, Hayatbini N, Fatemi MJ (2017). Body Image Coping Strategies among Aesthetic Surgery Patients in Iran. World J Plast Surg.

[29] Zakavi AA, Marzband R, Moallemi M, Mohammadpour RA, Afzali MA, Mohammadi Laeini MB (2015). Level of Spiritual Health According to Islamic Teachings in Students Studying in Mazandaran University of Medical Sciences. Journal of Mazandaran University of Medical Sciences.

[30] Rahimi N, Nouhi E, Nakhaee N (2014). Spiritual health among nursing and midwifery students at kerman university of medical sciences. Journal of hayat.

[31] Rehman R, Syed S, Hussain M, Shaikh S (2013). Health and spirituality ‘walk along’ in wellness journey of medical students. J Pak Med Assoc.

[32] Yuen CY (2015). Gender differences in life satisfaction and spiritual health among the junior immigrant and local Hong Kong secondary students. international Journal of Children’s Spirituality.

[33] Deb S, McGirr K, Sun J (2016). Spirituality in Indian University Students and its Associations with Socioeconomic Status, Religious Background, Social Support, and Mental Health. J Relig Health.

[34] Crerand CE, Sarwer DB, Kazak AE, Clarke A, Rumsey N (2017). Body Image and Quality of Life in Adolescents With Craniofacial Conditions. Cleft Palate Craniofac J.

[35] Brytek-Matera A, Donini LM, Krupa M, Poggiogalle E, Hay P (2015). Orthorexia nervosa and self-attitudinal aspects of body image in female and male university students. J Eat Disord.

